# A diarized journey: an interpretative phenomenological analysis of the older person’s lived experience of a hip or knee replacement within a fast-track programme

**DOI:** 10.1186/s12877-023-04276-4

**Published:** 2023-09-25

**Authors:** Marisa Burger, Francois Watson, Annemarie van Wyk

**Affiliations:** Quality in Nursing and Midwifery (NuMIQ), Faculty of Health Sciences, North-West University, 11 Hoffman Street, Potchefstroom, 2531 South Africa

**Keywords:** Osteoarthritis, Total knee or hip replacement surgery, Fast-track programme, Older person, Interpretative phenomenological analysis

## Abstract

**Background:**

For the older person living with end-stage hip or knee osteoarthritis, a hip or knee replacement can be a traumatic event, influencing the physical, physiological, psychological, social and economic facets of daily living. This interpretative phenomenological study aimed to reveal and interpret the daily lived experiences of the older person before, during and after a primary total hip or knee replacement surgery in a fast-track programme in South Africa.

**Methods:**

A qualitative interpretive phenomenological study, collecting data through solicited diaries and reflexive member validation interviews from seven participants aged 65–75 years, who underwent a primary elective hip or knee replacement surgery. The surgical fast-track process and data collection process happened simultaneously. Data collection from the solicited diary started before surgery, continued during the surgery process and finished six weeks after surgery and this was followed with reflexive member validation interviews. Interpretative phenomenological analysis was used throughout the three phases of the fast-track programme.

**Results:**

Three superordinate themes developed during the three phases of surgery: “The holistic impact of pain on daily quality of life”, “Finding ways to cope”, and “Transition between independence and dependence and back”. Although former research confirms the physical impact of osteoarthritis on the older person and the success of fast-track programmes for subsequent hip and knee replacements, this study contributes to the holistic impact of the surgery on participants’ daily lives. The diarized journey of individuals through the psychological, psychosocial, physical, professional, and spiritual experience are described and interpreted in this study.

**Conclusions:**

Across the solicited diaries, it was clear that pain as catalyst impacted the daily activities of the individual physically, psychologically, and psychosocially. Pain was subjectively present at different intensities during all the phases of the replacement surgery, impairing mobilization and triggering roller-coaster emotions. In order to cope with physical and emotional difficulties while preparing and adjusting to the environment, participants reflected on social support, physical and professional support, and spirituality. Throughout the preparation, hospitalization and the recovery process, the transition between independence, dependence, and back to independence was significant, reinforcing the individual’s determination to recover.

**Supplementary Information:**

The online version contains supplementary material available at 10.1186/s12877-023-04276-4.

## Background

Globally, hip and knee replacements due to osteoarthritis (OA) are effective, life-improving, and frequently performed surgical procedures [1] Fortunately, medical knowledge and techniques have improved significantly since the first hip replacement in the 1960s [2] and the development of the total condylar knee replacement more than 40 years ago [3]. Implementation of a multidisciplinary and multimodal approach enhanced surgical techniques dramatically for major surgical procedures, including hip and knee replacements [4]. This approach developed as a quest to reduce the risks of complications after surgery and has laid the foundation for Fast-track programmes (FTPs) [4]. For this study purpose, different programs have been collective named FTP's. It developed from the Enhanced Recovery After Surgery (ERAS) concept, pioneered and developed by Hendrik Kehlet and a similar approach developed by Kerr and Kohan, namely the rapid arthroplasty mobilization protocol (RAMP). A FTP is known for its patient participation and multi-disciplinary participation and a series of pre- and intraoperative interventions to optimize health and improve postoperative recovery and discharge [4, 5]. Fast-track programmes have proven to be effective, with positive outcomes and high patient approval, and without increased complications [6]. The FTP also seems suitable, safe and viable in a South African private hospital setting for total hip replacement (THR), enhancing service delivery [7]. For older, fragile and high-risk patients undergoing THR and total knee replacement (TKR), pre-operative information and family involvement, multidisciplinary intraoperative techniques and reduction of stress reactions postoperatively, can all result in good outcomes [4, 8]. Reduced length of hospital stay, as well as cost and time savings, are some of the numerous benefits found in a study in South Africa [9]. From a nursing perspective, an orchestrated FTP delivers continuity of care and a sense of security for the patient and health professional, permitting the patient to take responsibility for his/her own healthcare progress [10].

Patients scheduled for THR or TKR surgery experience OA symptoms like pain, activity limitations and decreased range of motion, with an increased risk for obesity and comorbidities and, additionally, lower health-related quality of life and economic strain [11, 12]. Considering the symptoms of OA, the impact on the older person is experienced even more severely. As age increases, mental and physical capacity gradually decreases, intensifying the risk of illnesses and disease due to a variety of biological and life events affecting the older person’s health status [13]. For many older persons, the contributing dynamics between age and decreased muscle strength and muscle power, lowered physical performance and the fear of falling, contributes to a decline in quality of life [14].

In a replacement surgery programme with positive evidence-based clinical outcomes, the daily lived experience of the older person before, during and after surgery can easily be overlooked. In this IPA study, the older person is considered a self-reflective holistic being, actively involved and interpreting the traumatic experience of a TKR or THR physically, spiritually, emotionally and socially [15, 16]. However, compared to an unexpected lower limb fracture, the traumatic experience can be lessened during the preoperative phase. The preoperative period is a vital part of FTP’s and comprises of evaluations to identify any comorbidities, organ malfunction, nutritional status [17], or social challenges that may postpone postoperative recovery, along with psychological preparation by staff [18], relative involvement and education on expectations toward a shared set of recovery goals [19]. Cheng et al. [20] emphasized the need of the older patient to adjust to a new body and reclaim life while experiencing, sharing and journeying a lower- joint-replacement surgery. A study by Gustafsson et al. [21] provides useful insight into the older person’s process of being in control of their lives and the transition before, during and after surgery. This transition is illuminated in six steps—dreaming of a pain-free, able body again; fears just before surgery; a renounced body during surgery; an incapable body after surgery; adjusting to their body; and then finally an integrated re-embodiment. A follow-up study concludes that the operation is seen as an all-embracing life event with reflections on previous care experiences influencing fear intertwined with hope for the future and relief after surgery [22]. Recent qualitative studies highlight the effects of a person-centred approach to optimize care [23] and the effect of surgery on the individual [24], which are aspects easily overlooked before, during and after TKR and THR surgery.

The aim of the study was to reveal and interpret the daily lived experiences of the older person’s before, during and after a primary total hip or knee replacement, in a fast-track programme, using a solicited diary. The study was conducted during the three phases: preparation for surgery, hospitalization, and the recovery phase. To authenticate the experiences of the participants, the primary author used reflexive member validation interviews. Furthermore, the primary author’s interpretation activity, also known as the “double hermeneutic” process in IPA, validated the information. The surgical context that framed the older adult’s daily lived experiences was within an FTP, at a selected private hospital in the geographical area of Gauteng province, South Africa.

## Methods

### Study design

A qualitative IPA study design within an interpretivist paradigm, guided by the philosophical underpinnings of phenomenology, hermeneutics and ideography [25] was used. According to Smith, a twofold hermeneutic approach entails reading, writing, and reflecting on the phenomena within the context while the researcher conducts research while attempting to make sense of the participant's sense-making [15, 16]. The diary offers the chance to record events, feelings, and impressions in accordance with what is deemed a priority for him or her as each individual interprets a situation and finds a method to deal. It also helped ensure that daily routines were disrupted as little as possible and eliminated the possibility of researcher bias, all of which increased its reliability.

Since the requested journal had to be written out, the participants had plenty of time to think about the questions, add their own stories to the data, and decide how much of their personal information to reveal. Within this approach the aim is to grasp a deeper understanding of the importance of different individuals’ experiences as self-interpreting beings while providing accurate descriptions of both the experiences and responses thereof.

### Participants (sampling and recruitment)

Following ethical approval from the Health Research Ethics Committee of the Faculty of Health Sciences of the North-West University, (NWU-00308–20-A1), participants were purposively selected. Eight participants were approached and consented to participate in the study, of which only seven completed the diaries and were included in the study. Five participants were female and two were male, all aged between 65 and 75 years. Five participants underwent a THR and two a TKR. Potential participants were listed to undergo either a primary total hip or primary total knee replacement surgery at the selected private hospital with the same orthopedic surgeon, between April 2021 and July 2021. The designated 220-bed private hospital in Gauteng province, has ten theatres, and provides a wide range of medical and orthopaedic services. About 30 to 40 patients per month are receiving replacement surgery by the specific orthopaedic surgeon. Inclusion criteria were 65 to 75 years old and able to write in a diary without any additional physical exertion, in either English or Afrikaans. Potential participants were excluded if their surgery was cancelled, a contra-indication to the elective procedure from the physician or other unanticipated cause arose and if they tested positive for Covid-19 before surgery uptake. Of those that showed interest in the study and consented to participate, only one potential participant was excluded.

After gaining institutional permission (from the hospital), recruitment and enrolment of participants occurred in parallel with the surgery-management procedure. The practice manager, through in-kind permission and a confidentiality agreement, served as an Independent Person (IP) for the study and was responsible for the recruitment, enrolment and informed consent process, and was favoured for this role because of the trust and confidentiality relationship with patients formed in the private practice.

### Invitations/ recruitment

An invitation to participate in the study was extended during the first contact session (to plan the surgery) between the practice manager and the participants. The practice manager served as the IP with the only access to the list of potential participants, to cut out the possibility of conflict of interest and bias.

### Informed consent process

With the inclusion and exclusion criteria in mind, the complete study process was verbally explained by the practice manager, with guidelines on completing solicited diaries. The practice manager acted as the IP for the study and took consent as indicated during the same time period surgical consent for the procedure was taken. Informed consent for the study was signed by both the IP and the participant at the practice where the surgical consent process was completed.

### Data collection

Data were collected from two sources; the main source was a solicited diary and, at a later time, telephonic reflexive interviews were done to augment the data collecting process. Data collection happened simultaneously with the surgical process. Throughout the process of data collection, the primary researcher maintained a reflective journal for supplementary notes, exploring deeper interpretations.

In-depth or semi-structured interviews are the data collection approach predominantly associated with IPA [26]. The use of solicited diaries in more recent IPA research [27, 28] has indicated a great value, in that it allows the participants enough room to reflect on their life experiences [16] and express these through written language and within their cultural context [29] without limiting their time to reflect and think about the experiences [27]. Within this approach, writing a solicited diary was a conscious decision made by the researchers. Diaries seem to provide an excellent alternative to providing a narrative account for analysis [30], reducing the possible bias and influence of the first author and assisting in the minimum disruption of daily activities without any intervention from a health professional, making it more reliable. Diaries or other additional tools also help to facilitate understanding between the researcher and participant [31]. It took time to write the solicited diary, giving the participants enough time to reflect on the questions and enrich the data with their personal narratives and allowing them to control how much information they wished to share. Throughout the process of data collection, the first author maintained a reflective journal for supplementary notes, exploring deeper interpretations. A pseudonym was designated to each participant and identifying details were removed.

Each participant received an A5 paper booklet with semi-structured questions and guidelines for the solicited diary. diary (see Additional file 1). A pseudonym was designated to each participant and identifying details were removed. Participants were encouraged to daily reflect on their physical, emotional, social and spiritual experiences, for at least 5–10 min, before surgery, during hospitalization, and for the six weeks recovery. Most participants started the solicited diaries five days before surgery (the longest being 20 days before surgery) and returned it at the latest 21 days after completion. As they progressed through the FTP, the participants had the chance to record events, emotions, and thoughts by filling out the requested diaries in accordance with what they considered to be a priority. Additionally, it made sure that everyday routines weren't too interrupted and removed the chance of researcher bias, all of which improved the data's objectivity.

Not all participants preferred writing in the provided booklet, and some opted for electronic entries. Four participants completed the solicited diaries with handwritten notes while two made voice and written notes on their mobile phones and another participant typed notes reflectively. In preparing the data, the primary author doing the fieldwork typed a verbatim record of each diary. This was done in table format with columns and rows, according to daily notes made by the participants. Two diaries were written in Afrikaans while five were written in English.

Telephonic reflexive interviews were done by the primary researcher, after analysis of each diary, supplementary to the solicited diary. These interviews on average lasted 45 min and were followed by peer debriefing meetings between the authors continuously during the process. The longest interview was 53 min and the shortest was 38 min. Initial themes from the solicited diaries guided the reflective, semi-structured questions during these interviews. Further probing questions facilitated the composing and clarifying of the participant’s lived experiences. During the conceptualising phase of the study, the researchers based on Smith and Osborn’s [32] suggestion and other similar studies aimed to include, six (6) to nine (9) participants in the research. During the data collection and analysis phase, the participant cut-off decision of eight (8) participants was guided by an adequate contextualisation of the older persons' experience throughout the FTP (Table 1).

**Table 1:** Demographic information of the participants

Demographical and Biographical Information							
**Pseudonym**^a^	*Cathy (P1)*	*Betty (P2)*	*Susan(P3)*	*Mark(P4)*	*Richard(P5)*	*Sofia(P6)*	*Florence(P7)*
**Gender**	Female	Female	Female	Male	Male	Female	Female
**Age**	70	68	70	69	75	74	66
**Living arrangement**	House	House	House	Residential complex	Retirement village	Retirement village	House
**Marital status**	Widowed	Married	Life partner	Married	Married	Married	Married
**Language**	English	Afrikaans	Bilingual	English	Afrikaans	English	English
**Type of surgery**	Right TKR	Right THR	Left THR	Right TKR	Left THR	Right THR	Left THR

^a^All participants’ names are pseudonyms and not their real names

### Data analysis

The data analysis was interpretative and used the seven-step technique of Charlick et al. [26] as developed from the work of Smith and colleagues [15] for analysing each solicited diary from an IPA perspective. This process of analysis extended over months since each diary was collected at a different time. The process involved the reading and re-reading of each solicited diary; reflection and making notes, identifying and developing themes, and finding patterns for each diary. In order to stay true to the hermeneutic circling movement of IPA, the primary author doing the field work had to constantly return to the raw data, challenge her own assumptions echoing in the mind, and illuminate the participant’s reflections and not the author’s. The second part of the analysis started as an intertwining step after the fifth step for each diary. The same technique was followed for the analysis of the reflexive member validation interview. Notes were made in the reflexive journal while exploring the content of each diary and each interview.

Due to the role of the primary author doing the fieldwork, as a nursing professional in the orthopaedic practice, there was a deep understanding of the issues and problems affecting participants.

### Trustworthiness

To ensure trustworthiness, the criteria of Lincoln and Guba were applied Lincoln and Guba [33] and Botma et al. [34]. Prolonged engagement with the diary, using two data sources and repeating the steps for the reflexive member validation interview contributed to the criterion of credibility. Additionally, regular contact sessions via Zoom with co-supervisors for peer debriefing contributed to the trustworthiness of the study. For the criterion of transferability, purposively selected participants were chosen and thick descriptive data according to IPA steps for reading and re-reading and reflection of analysis were used. However, due to cultural differences and personality traits, congruence might not be achieved in another study. To accomplish the criterion of dependability, an audit trial was kept with dense descriptions in the reflective journal alongside the data analysis. The criterion of conformability is accomplished with the reflexive validation interview supplementing triangulation and with-in method triangulation to validate reflection within two data sources.

## Results

Across the solicited diaries and reflexive member validation interviews it was clear that pain was a pervasive catalyst at different intensities during all the phases of the replacement surgery. Pain impacted the physical, psychological and psychosocial daily activities of participants and is considered the essential stimulus. For this reason, one of the superordinate themes prevailed as the holistic impact of pain on daily quality of life.

As the participants journeyed through the surgical process, different physical and/or professional support as well as psychological and psychosocial support became part of coping. For some, spiritual coping mechanisms were part of life. Finding ways to cope persisted before, during hospital stay, and during recovery and is therefore considered a second superordinate theme.

All participants encountered some degree of transition between independence during the preparation phase which changed to dependence on people or resources during the hospital stay phase. During recovery, it transitioned gradually to independence again. This transition was not so explicitly reflected on by the participants but persisted as a superordinate theme throughout all the phases.

These three superordinate themes with the supporting subordinate themes as revealed through the data analysis are summarised in Table 2 below followed by an in-context reporting on the superordinate themes with representative quotations.

**Table 2:** Three superordinate themes with seven subordinate themes, during three phases

***Different phases***	***Superordinate Themes***	***Subordinate Themes***
**• Preparation for surgery** **• During hospital stay** **• During 6 weeks recovery**	**1. The holistic impact of pain on daily quality of life**	*The psychological impact on daily life*
		*The physical impact on daily life*
		*Psychosocial impact on daily life*
	**2. Finding ways to cope**	*Physical and/or professional support*
		*Psychological and psychosocial support*
		*Spirituality as support mechanism*
	**3. Transition between independence and dependence and back**	*Independence and dependence*

### The holistic impact of pain on daily quality of life

Before surgery, participants realized the irrefutable emotional impact the pain and deteriorated quality of life had on their daily lives. The inability to walk as before and complete tasks they were previously able to do, convinced them to proceed with surgery. Through these physical difficulties and emotions, some participants conveyed the importance of social connectedness and support to persevere with surgery. Directly after surgery in hospital, participants’ pain was immersed in emotional irritability in an unfamiliar environment. Physical pain was intense and other people, hospital staff or equipment either contributed to contentment or more frustration. At home, slow progress and continued pain caused despondence for some participants while others were relieved at the difference in pain, compared to before surgery. Daily physical progress was evident in all diaries during the six weeks. Gratitude was expressed as interaction with family members, friends or physiotherapists continued.

#### During preparation for surgery

During the preparation phase for surgery, seven participants experienced an inability to continue with normal activities, with pain adversely affecting their daily lives psychologically, physically and psychosocially. Since the interconnectedness of these factors refers to the wholeness of the human person, quotations are closely related.

##### Psychological impact on daily life

Six participants indicated a daily emotional roller-coaster, wavering between anticipation and nervousness while preparing for the unknown. Betty, three days before surgery wrote*: “I feel depressed and realize there is only one way out—surgery needs to be done”. (P2),* while Sofia conveyed similar emotions five days before surgery “*Over the last few days I have felt down at times, and forgetful – then realized that I have been feeling stressed underneath. But at the same time positive about the outcome of the operation”. (P6)* Living with pain also presented an emotional fluctuation between feelings of depression and a readiness to be relieved of pain, while at the same time envisaging positive outcomes. Richard wrote six days before surgery, “*The thought of how it will feel without pain is truly motivation for me to be courageous”.* Feelings of anxiety were expressed repeatedly by many participants, reinforcing the need for surgery. One day before surgery Cathy wrote *“I am looking ahead to no more pain, and that is keeping me positive”.*

##### Physical impact on daily life

For some, the physical struggle to walk and the prospect of a pain-free life provided motivation for surgery. Daily activities and quality of life were diminished, triggering rational decisions to resolve the pain. Sofia expressed herself five days before surgery *“Today I’m feeling tired of the pain and having to hobble around, and using a walking stick to give me some balance and confidence that I won’t fall again”*. For seven participants it was not a sudden disability but a gradual, amplifying physical struggle. Betty was clear in her diary; *“We went to town and getting in and out of the car became too much afterwards. The pains trouble me”. “Dinner is prepared and the dancing around the pots are tiring. I try to carry on with all the pain in my groin and hip”.*

##### Psychosocial impact on daily life

The effect of pain and impaired mobility influenced daily activities and, indirectly, social dynamics. Reflections on the feelings of acceptance, support and social connectedness were mentioned by two female participants, thankful for the social support of family. Sofia diarized four days before surgery *“I feel good having an afternoon when I can be on my own with Jamie (the cat)! And I look forward to my husband’s return and a lovely evening eating supper he has prepared, and we’ll watch something on TV”.* Additionally, the support of animals was diarized by both. Cathy wrote: *“What would I do without my family and friends? They keep me centered and positive through all this. It can be hard to come home to an empty house. Thank goodness for my wonderful dogs, they just shower me with love”. (Day 4 before surgery).*

#### During hospitalization

Pain was expected after surgery. However, for a few participants, pain was abstruse, triggering nuances of emotional irritability, annoyances and frustrations with things and people.

##### Psychological impact on daily life

This had an extensive emotional impact on their hospital stay. Cathy reflected on the day of surgery *“Emotionally – I wished I had never gone down this path*” while Susan wrote, *“The nurse came and woke me up to do the normal wash at 23.00 and I was very rude in telling her that she must just leave me to sleep”.* Day one in hospital Sofia diarized*, “Someone needs to invent hospital beds that are patient-friendly and not only hospital-staff-friendly (as well as bedside cabinets)”.* For Cathy, pain persisted and on Day 3 she wrote *“I try to push through it to do the exercises, but it’s hard and there are only so many pain signals I can ignore”.* Mark contemplated on hospital stay as an anticipation to return to what was considered normal to him*, “I was praying that I would make it to Saturday 11am, when discharged back to normality”.*

##### Physical impact on daily life

On the day of surgery two participants diarized their physical pain as intense. Cathy wrote, *“I would never [have] imagined such pain. They were a long time coming and I was in agony. I don’t think I slept a wink with all that pain. The meds didn’t help at all”.* Sofia wrote, *“The first night was awful. I was in such pain. My heel (the op side) was in agony. Night staff didn’t seem to know what to do. Gave me Paracetamol. Slept and woke very unhappy and still in pain”.* For another participant, the environment repeatedly attributed to pain as Richard remarked on Day 1 and Day 2, *“Mattress causes a lot of discomfort which contributes to pain*”, and “*After a successful restless night on a mattress that needs a match, I am still alive and very grateful that I am no longer burdened by the pain I had to go through before”.* The last part of his remark, however, reflects on a change in his pain experience, conveying relief.

##### Psychosocial impact on daily life

Irritability was expressed by a few participants regarding staff members or other patients on the ward. Although not specifically mentioned as intensifying pain, these aspects had a psychosocial impact on the daily experiences during hospital stay. Mark reflected on his hospital stay, *“Most of the nurses were 100%, unfortunately the ones lower down the pecking order were loud, very loud, they have no issues in the middle of the night, bumping your bed with a trolley or another bed. The Ward is chaotic. The nurses shout at each other although stood next to one another. Each patient has a buzzer to summon a nurse, these buzzers are going off every 5 s so sleep, is very difficult. On top of that, the beds are soft and a bit sunken, so getting into a restful position is almost impossible”.* Richard commented in a positive and thankful manner to his experience with staff on the day of surgery*, “During the night, a nurse was constantly present to monitor the situation. I would like to thank [the male nurse] X [name withheld to ensure anonymity] because he really supported me a lot.”* Sofia diarized the positive psychosocial connection with other ward patients on Day 2. *“I enjoyed the company of the 3 other ladies who were in the ward with me. Interesting that one forms a kind of bond”.*

#### During six weeks recovery

All participants had unique experiences within their own environment and context. Pain was entangled in all aspects of life and influenced their daily awareness of activities.

##### Psychological impact on daily life

Reflection on pain during the recovery time extended over six weeks, meaning it was not a once-off reflection but a continuous process with continuous changes. Cathy wrote on Day 1 at home, *“This knee replacement has changed my day-to-day life, by filling it with pain. I know it’s early days but I don’t want to do things that will hurt”* while on Day 4 she wrote *“I get very depressed sometimes with no end to the pain”.* On Day 15 she was determined to continue with exercises despite pain*, “I am doing the exercises until the pain makes me stop. I have gone through too much to sabotage it by stopping the exercises”.* Much later, only on Day 35, did she reflect in a different way on the emotions she experienced the first few days, *“In the beginning I was in so much pain that it was hard to move and the exercises nearly impossible. Doing even the smallest thing was a major effort. I was depressed quite a lot. The stockings were a huge trauma in my life. It was only when I took them off for good, 10 days after the op that some of the depression started to clear and I finally felt as though I was starting to heal”.*

For another participant, Betty, the pain of the replacement became insignificant when she experienced a setback. She wrote on Day 3, *“No improvement to numbness and pins and needles. I have already prepared myself psychologically to be patient with this. It has an influence on my mood. If I could choose, I wouldn't have it”*. The psychological impact was immense, influencing her emotions and relationship as she wrote on Day 14, *“My foot is very sore and stiff and swollen. My emotions run high and I shed a few tears of discouragement. I am impatient and my poor husband is suffering”*. The pain she experienced was caused by a blood clot, as discovered upon examination by the surgeon. This obstacle in her recovery increased the impact of pain in her life but was not directly related to the surgical pain. On Day 26 she wrote, *"I try to keep up my spirits but I always find myself in a struggle and fighting over all the pain, inconvenience and discomfort"*.

On Day 4 Richard realized his restriction related to surgery but diarized a grateful liberation of being without the disabling pain: *“It's harder to accept that I'm limited because of the replacement, but importantly, I have to be careful with what I do. I am of course delighted that the pain that has hampered me for a long time is finally no longer there”*. Florence, who was a Comrades Marathon runner, was grateful to be relieved from joint pain and continued to be thankful. On Day 1 she wrote, *“I am so happy to be home and not have the joint pain any longer”,* as on Day 37 she diarized: *“Shared with (friend) X (name withheld to ensure anonymity) that I absolutely recommend the surgery, it is such a relief not having a Toothache pain in my hips. I am pain free and so thankful”*. At a later entry, on Day 41, she reflected on a nostalgia she experienced while mourning and rejoicing: “Now that the healing is sort of complete, I feel depressed when I see runners or when we drive on our running routes. Thankfully I can walk, cycle and hike”.

##### Physical impact on daily life

Pain was persistent but gradually subsiding, initially causing a decline in daily activities. On Day 2 Cathy wrote, *“I have to admit I increase painkiller dose; I didn’t want to get out of bed because it hurt so much”.* By Day 17 great improvement was noted*, “Every day I am managing to do more things for myself. I am starting to walk with one crutch sometimes. It’s a bit painful but I can do it”.* Continuous pain was experienced but the anticipation to be pain-free was motivational on Day 2: *“It doesn’t seem like I will ever be pain free again, but I just have to trust that it will come right eventually*”. For Betty, the hip pain initially experienced subsided on Day 1 and she wrote: *“What I can say is that since the surgery, the pain in my groin is gone. I endure pain and attribute it to the operation—there is a cut and swelling in the limb”.* On Day 10 at home, she noted *“The operation part feels good although I still experience discomfort”*. As Mark reflected on his pain journey, he diarized a continuous pain: *“The pain has not receded and won’t for another 2 weeks or so”*.

As time passed, Florence expressed her gratitude on Day 13 for pain-free mobility: *“The post operation discomfort is such a small price to pay now that I have pain free mobility”*. For her, the pain shifted to discomfort on Day 16, *“I have no pain where the surgery took place only discomfort”*. Richard agreed with her on Day 20, *“Surprised again when right hip has no pain. Wonderful. I walk well which brings great relief*”.

Daily activities were influenced for Richard when he diarized at Day 23*, “A replaced hip doesn't like getting in and out of a car very much. This causes a dull pain that takes a lot longer to subside”* while Florence on Day 28 noted, *“My days are normal and pain free and my mobility is improving”*. Gratitude to be pain-free was also expressed by Sofia on Day 39, *“I am so grateful and pleased that Dr did the procedure and now I have new hip, which I know will make a huge difference to my life and enable me to be able to walk without pain”.*

##### Psychosocial impact on daily life

Having people around at home was noted by some participants in a positive way. Help, support and optimism was influencing the daily emotional progress, as Cathy reflected on Day 7 when the physiotherapist intervened*: “My physio has just left. She is very happy with my progress. Swelling is less, range of motion is more. She added another exercise and did a laser treatment. She is happy with my walking and going up and down steps. That’s all good news so I am happy as well”.* For Betty the assistance of her husband was paramount as she depended on him by Day 5, *“The wound is cleaned by my husband. I won’t be able to do it myself. I cringe at the sight of a wound, it's a good thing I never became a nurse/doctor”*. Florence also expressed amusement on Day 13 with her grandchildren as she recovered, “*My grandsons are such a pleasure to have around and they cannot believe I had another hip replacement, because I look normal and I am mobile”.*

In summarising the holistic impact of pain on daily quality of life, the findings emphasize the psychological, physical, and psychosocial impact of pain during preparation, hospitalization and during the six-week recovery period. Emotional fluctuations and anxiety accompanied by reduced daily activities due to pain, had reinforced the need for surgery. During the hospital stay, pain was overwhelming with additional discomfort in a new environment with other people and staff. During the six-week recovery, reflections on pain varied by participants but gradually diminished as mobility improved. The psychosocial influence was more noticeable during this period.

### Finding ways to cope

Finding ways to cope with pain and restrictions is a natural way to endure life. For some participants it was not explicitly mentioned but unfolded in daily notes as their journey developed/advanced. To deal with daily life, support from different people and means revealed itself. The use of physical devices like crutches or painkillers as well as the professional support they received before, during and after surgery was meaningful to them. Psychological strategies like self-talk and self-motivation as well as psychosocial support from family members and friends was another way to cope. For some participants, their spiritual connectedness helped them to tolerate the difficult times and carry them through.

#### During preparation for surgery

Five participants tolerated pain and restricted activities before surgery but with different intensity and time intervals. However, to manage daily activities and continue with life until the surgery, everyone was finding ways to cope. These coping strategies consisted of physical and/or professional support, psychological and psychosocial support and, for some, spiritual practice.

##### Physical and/or professional support

The use of pain killers and walking devices, as well as the educational session and information booklet as part as support from the doctor’s rooms, were considered useful coping mechanisms. Physical ways to cope included the use of medication, as Florence noted 14 days before surgery, *“I am so depressed and have to continue medicating with Tramecet pain killers … And this is affecting kidneys.”* Betty also used painkillers as she noted three days before surgery, *“I take pain medication, although it does not completely take away the pain”.* For Cathy, the information discussion was disturbing, and she wrote six days before surgery, *“I was feeling quite upbeat this morning until the phone discussion with sister (practice nurse) X (name withheld to ensure anonymity) I know she has to do it, but talking about all the things that can go wrong put a damper on my mood*.” The education session was perceived differently by Betty as she was comforted by the information and the telephonic session five days before surgery. The day before she left home when she diarized, *“Sister (practice nurse) X (name withheld to ensure anonymity) calls me and explains everything about the operation to me. I feel more at ease about the operation after her conversation with me”. I have studied the documents carefully regarding the operation, aftercare *etc*. and feel reassured for what lies ahead.”* Sofia used a walking stick to cope, as she noted five days before surgery: *“… using a walking stick to give me some balance and confidence that I won’t fall again”.* Mark remarked on the initial consultation with the doctor, showing his appreciation, *“He gives me a hearty laugh and pats me on my shoulder and tells me not to worry, it will all work out fine. He books my surgery for the following week. I’m happy I’m dealing with a Human Being and not a Cash Register”.*

##### Psychological and psychosocial support

Feelings of acceptance and social connections contributed to psychological satisfaction, contributing to a way to cope. Psychosocial support was expressed in relationships with family members like a spouse or daughter, or the assistance of a domestic worker. Some only mentioned the presence of a partner without focusing on the support received from them. For Cathy, her daughters encouraged her five days before surgery as she wrote, *“I woke up very depressed this morning. Too much time to think about what’s coming. My daughters reminded me that Jesus is always with me, and managed to get me cheered up*”. Her domestic worker was also a great assistance to rely on, “[Domestic worker) X (name withheld to ensure anonymity) *says she is ready to do anything that has to be done. I have complete faith in her*”. Sofia had a social support network which she could count on and felt part of: “*Others who stopped by, wanted to know all about the operation I am having next week. It feels nice that they are interested*”.

##### Spirituality

Three participants found spiritual practice a way to endure uncertainty and anxiety before surgery. A spiritual reassurance and convincing faith were easing their nervousness and spiritual friends contributed to a feeling of connectedness. Four days before surgery Sofia found solace as she wrote, *“I have faith that Jesus, God and the Holy Spirit will be with me every moment through the op and afterwards, although He never leaves me anyway*”. Richard experienced a calming effect as he noted three days before surgery, *“The morning church service (message) is very encouraging and helps to calm my peace of mind, knowing that within a few days there will be relief with new prospects”.* The day of surgery Cathy also prayed for consolation, “*Going to the hospital just now. I am trying to keep the sense of calm and peace I had yesterday. I will keep praying for peace*”.

#### During hospital stay

While in hospital, six participants depended for a period on professional people to come to grips with their circumstances. The support of people during the short interval before surgery made an impact on a few participants. Furthermore, psychological and social support from others, and the spiritual connectedness some participants had, was part of coping with the new environment and adversities.

##### Professional support

Being in a new environment, participants were finding ways to cope with the physical disability directly before and after surgery. Professional people had a significant influence on the participants to ease them into a mental calm before surgery, as diarized. Just before surgery Cathy noted: *“I had an epidural injection before. I was put under by Dr X, the anaesthetist,* (name withheld to ensure anonymity*) was friendly and reassuring”.* Mark noted: *“The anaesthetist was a delight, good humoured and I loved his banter”.* Mark also remarked on an inconvenience for him in hospital routine: *“For some reason, only known to the few, you are woken at 5am or earlier by the physio*”. To Richard the professional support was noted as remarkable: *“Very restless night. But thanks to* [the male nurse] X [name withheld to ensure anonymity] *who assists us in ward 7”.* Florence recalled advice two days after surgery, *“The pain in my groin subsided quite a bit. I remembered what my anaesthetist said, don't chase the pain, get medication before the pain”.*

##### Psychological self-support and social support

A self-reflection on intrinsic motivation and mindful self-talk became part of finding a way to cope. Writing these down in a diary as part of a monologue, is related to mental self-support. On Day 1 in hospital Betty wrote, *“I motivate myself—bite the bullet and push through”.* Cathy encouraged herself when she diarized, *“I am going to try some exercises before supper arrives”.* Susan wanted something she was familiar with and had psychosocial support from her partner: *“My partner brought me Cranberry Juice and Ricoffee that I used while in the hospital”.* For Richard the support of his son was noted, *“When I regained my composure, my son was with me and encouraged me”.* Although not explicitly mentioned by participants, social support was important for participants. Sofia contemplated on Day 2 in hospital as she diarized, *“Thinking about it – I realized that I’m often helping others, and this was a time when I have to receive help. It’s not as easy. It is more humbling having to receive graciously. It became obvious that there are so many things that we do automatically and not need help with. Example: Drop a piece of paper, pea or whatever, and you can’t pick it up! So I will carry around a braai tongs, and that helps!”*

##### Spirituality as a support mechanism

For some participants a spiritual belief and connection brought assurance and eased anxiety before surgery. Both Cathy and Sofia noted on the day of surgery, *“Having been through times like this before, I know that God is on my side”. Sofia wrote, “I was not afraid. I committed myself to God”. Florence also thought of other as she diarized, “My prayer was for God to guide the hands of everyone responsible for my surgery this morning”.*

#### During six weeks recovery

As individuals, each participant went through a unique recovery period of discomfort, pain, and difficulties. These hardships can only be understood in the recovery process diarized by each individual. Since each individual's experience was unique, this study took the decision to deal with each participant’s recovery process in its entirety—something not done in the other phases. The professional support received bolstered not only their coping abilities but also their self-ability to manage.

##### Professional support

The physiotherapist played an immensely encouraging role in the physical assistance to help participants cope and recover. Richard went to a subacute facility for four days while all participants obtained support from physiotherapists. For Cathy this was noticeable on Day 8 of her diary, “*She is happy with my walking and going up and down steps. That’s all good news so I am happy as well*”. To physically be able to get around with less support as time progresses, was highlighted in the notes on Day 13: *“I was in the bathroom, my mind a million miles away. Suddenly I realized I was walking without my crutches. I was surprised! I got washed and dressed without help and that has given my confidence a boost.”* Knowing one’s limits and own physical strength was also noted on Day 25: *“I find the exercises easier each day, but it still hurts to sit with my knee bent. Getting out of a chair is painful. I am able to walk with one crutch okay, but eventually the pain pushes me back onto two”.*

##### Psychological self-support

Being able to figure out how to cope with the pain and disability was a step towards improvement for all participants, as noted by Sofia on Day 2: *“My leg that was operated on is slowly gaining strength. A very comfortable position, when getting into, or onto the bed is when I lift up both legs and bend them together. It’s a bit painful to lift my right leg in the morning, but it’s worth it when I can stay in this position for a while. I think the pain is just stiffness”.* Sofia’s self-advancement was diarized on Day 3*: “But I have achieved a lot, I am now able to walk unaided for the 5th day!! I stand up, lock my knees and wait for a bit, then take a step, then I’m ok. I do make sure that I can touch the couch, chair, table, wall *etc. *just to give myself a bit more confidence. But I can also walk from the kitchen to the patio, with coffee in one hand and chocolate cake on a plate and I get there without a problem!!”* Betty self-reflected on the pain in her foot on Day 3, which affected her psychologically, *"I have already prepared myself psychologically to be patient like this. It has an influence on my mood. If I could choose, I wouldn't have it. I was so looking forward to a "normal" foot, with no pains and discomfort”.* Self-encouragement and patience were needed on Day 7 as she diarized, *“I get my ‘ups and downs’ and take comfort in the fact that it is only 10 days after my operation and gain new courage”.* On Day 14 she experienced despair, *“My foot is very sore and stiff and swollen. My emotions run high and I shed a few tears of discouragement. I am impatient and my poor husband is suffering”.*

For Richard, on Day 3, the way to cope was in being organized: *“I have to organize my time so that I systematically complete my daily tasks. It's harder to accept that I'm limited because of the replacement, but importantly, I have to be careful with what I do”.* On Day 5 he highlighted the importance of self-talk: *"Up early and decided to follow a new way of thinking”.* On Day 14 he found a way to return to the able person he used to be when he diarized, *“Often, once I get going, I feel better. Furthermore, I concentrate on daily exercises and remain calm and composed.”*

##### Psychosocial support

Contact with and social support from friends and family contributed to wellbeing and a gratifying reassurance of their importance to others. Cathy noted on Day 8: *“When something like this happens you learn who your real friends are. I am lucky to have some really good friends”.* The presence of her animal companions elevated her spirits and she mentioned on Day 25: *“The day started as usual with my dogs, Mimi and Stanley, taking flying leaps onto my bed to say ‘Good morning’. They are so full of joy and love it’s impossible to feel gloomy with them around”.* For Betty, her husband’s support was reassuring as she diarized on Day 23*: “I can't do anything about it, I'm dependent on my husband and I'm just absolutely frustrated and done”* and on Day 32 she wrote* “I am just discouraged, and my husband encourages me”.*

For Susan the psychological effect of the replacement was noted on Day 3 as: *“Very depressed just stay in bed and cry. Eat very small portions of food but no appetite. Very dizzy”,* and on Day 4 she did not feel better yet. *“The children and grandchildren came to visit. Don’t want so see them. Very depressed”.* The psychological change was on Day 5, as she noted*, “I made a mind decision to get out of the depression”.* For Mark the reflection on recovery was significant in being able to return to normality. He wrote, *“Whilst lying in bed, recovering and the pain is unbearable, just count the days as sleeps, you have approx. 16 to 18 sleeps before you can start your recovery and back to a normal life. In less than a month you should be walking normally but slowly again, pain free”.*

The social support Sofia received was psychologically uplifting, as she wrote on Day 7: *“I am in touch with* (friend) *X* (name withheld to ensure anonymity) *who was in the ward with me, and she had a full knee replacement. She is very encouraging and I like to think I encourage her too. This helps a lot! I have lots of friends in the village and church here and outside, who often WhatsApp me and its very nice having so many supporting me”.* Betty relied on her husband when she wrote on Day 18,* “I am so grateful for my husband who supports and assists me so much. I can't cope alone at all”.*

On Day 3 Florence also mentioned the support from her family, *“It's a joy having my husband and grandson wait on me. Breakfast, lunch and supper”.*

##### Spirituality as a support mechanism

Four participants reflected in a spiritual manner, contributing to a supportive contentment with the situation in which they find themselves. On Day 6 Cathy diarized, *“From a spiritual point of view, I am doing fine. God and I are still on speaking terms. Sometimes I just rest in his presence like a child curled up on her father’s lap. No need for words”.* Her reflection on Day 23: *“If I didn’t have my relationship with God to lean on, it would be quite depressing”* and on Day 26 she wrote*, “I am feeling a bit depressed but I manage to kick myself out of it by some singing and praying and positive thinking. I know that God is always with me, what more do I need*”. Richard also found peace in a spiritual promise when he wrote on Day 3*, "At this stage, I am holding on to the promise of our Heavenly Father not to leave us or forsake us".*

Sofia expressed metaphorically what the connection to her cat and her spirituality as support meant on Day 23*, “This morning I am feeling stronger and I think a better night’s sleep has helped. We have an adorable black cat called James, Jamie for short. He has hardly left my side. All night long he has cuddled next to me, and he even lay on my arm and fell asleep. Incredible comfort. I believe the Word is showing His presence and support through this beautiful cat. He is on my lap as I write this, and we are warming each other!!”* Florence also expressed gratitude on Day 31, *“Thankfully I'm blessed, I have no pain after the hike, my joints are fine as well”*.

To summarise ‘finding ways to cope’, during these three time periods, the physical and professional support received was highlighted. The psychological self-support and psychosocial support revealed itself in relationships with others. Three participants experienced connectedness in a spiritual way, as a coping mechanism. During the hospital stay participants had diverse encounters with professionals who had a significant influence on their experience. Self-reflection brought new discoveries of their own ability to cope. The connection with spirituality was reassuring for some while in a difficult environment with adversities. During the six-week recovery, the help of the physiotherapist emerged as crucial while gradual self-support came to light with a sense of achievement. Psychosocially, family members were a great support to cope in the new environment. Throughout the recovery, spirituality became visible as a coping mechanism for some participants.

### Transition between independence and dependence

As evident from previous reflections, the phenomenon of a replacement brought personal adjustments in daily functioning. These are visible in the ability to function independently during preparation, and right through to the undeterred determination to return to independence after the period of dependence in hospital. The transition was gradual, with daily advances.

#### During preparation for surgery

As evident from the notes of participants, independence before surgery was unquestionable. Preparation, inspired by the educational session, was vital for five participants to be able to return to normal life after the surgery. Some participants mentioned the things they did and bought, like Cathy five days before surgery, *“I need to go and do a lot of shopping to stock the fridge and pantry for a month while I can’t drive. Will also make sure I have a supply of the dog’s meds and supplements. There is a lot to prepare and organize so that things run smoothly when I come home”.* Susan and Richard also diarized specific things they bought, “*Went to the pharmacy to get the aid for the house to be ready for the time after the operation. Bought the crutches, seat raiser, handrails for the shower and a nonslip mat for the floor in the shower. Loaned the walker from a friend that had the hip replacement 3 months ago”*. Planning was vital for Sofia on Day 3 of preparation, *“I was mostly getting things organized in the home and planning what to take clothing-wise to the hospital”.* Florence also prepared on Day 2 before surgery*, “Laundry, I'm making sure everything is washed before I go into hospital. Baking—savory muffins, banana and chocolate bars, enough for an army. I have to freeze half in my already overflowing freezer …”.*

For some participants the lack of normal functioning like driving to town for shopping or even watching TV and sitting for a while was strenuous. This is a gradual change to dependence, especially when the use of medication increases. Betty noted four days before surgery, *"Unfortunately, sitting for a long time is exhausting for me and sometimes I just have to get up and move around a bit".*

#### During hospital stay

Surgery changed participants’ reliance on others for basic activities. Although not always mentioned explicitly, this was noticeable in the journey through the diaries. Cathy noted on Day 2 in hospital, *“This morning I was able to walk, with a lot of help!’* Susan was dependent on others for basic needs, bordering on humiliation on the day of surgery, *“I got sick. This was an embarrassment to me as my bowl went at the same time and I had to ask for the staff to clean me as I was not capable to move my lower body after the epidural for the next 9 h*.” For Richard, being washed on the day of surgery was diarized since he was not able to do it himself, *“The bed bath was quite an experience as I have never experienced such an event”.* Even for an activity like walking, which Sofia was previously able to do, she needed help on Day 1 in hospital, *“Physio came and helped me to stand onto the floor. That was good. No walking this morning”.* A realization of the reliance on others came to her when she wrote on Day 2*, “It became obvious that there are so many things that we do automatically and not need help with. Example: Drop a piece of paper, pea or whatever, and you can’t pick it up!”*

#### During six-week recovery

Self-motivation and the determination to return to independent functioning were characteristics of this recovery period. The gradual progress to being able to rely on oneself to function independently again, brought satisfaction. Some grappled with disrupted daily routines, especially toilet routines, needing to go more often at night. One participant had a setback and experienced more dependency on her husband. The freedom to go shopping again was mentioned by one participant while another looked forward to drive again after the six-week follow up.

Cathy’s progress to independence was noted as she wrote on Day 1, *“Getting into my bed was another challenge, but I made it in the end”.* On Day 2 she wrote, *“I am feeling more independent and able to do things on my own more”* while at Day 11, *“I got washed and dressed without help and that has given my confidence a boost”*. When she was able to do laundry herself on Day 31 she diarized, “It was a real sense of accomplishment when I got everything on the line”. Betty had a setback and was not able to cope herself, depending on her husband when she wrote on Day 23, “I can't do anything myself, I'm dependent on my husband and I'm just absolutely frustrated and tired”. Basic daily activities that Susan was able to do before surgery gradually returned as she wrote on Day 30, “Started cooking and can help myself with the shower and dressing except of putting on my socks”. For Mark the transition to independence came when he was able to be at his computer, bringing anticipation for recovery, “By this time, I am only using one crutch, so I slowly mounted the stairs one by one. When I got to the top and sat at my computer screen once again, I knew I was on the road to recovery. Now I could go onto the Internet, read the papers online, write some emails, catch up on my work and watch Netflix! Yeah!” Sofia progressed from *“Relying on others for the smallest things is hard for me, I can walk to the kitchen with my walker and make coffee and put cereal and yoghurt on my fruit, but I can’t carry it to where I will be sitting”,* on Day one, to “I dressed myself today, very chuffed!!” on Day 12 and *“I am walking unaided around the house sometimes. As long as there are walls, tables, chairs *etc. *to touch of hold onto! I can bring a cup of coffee from the kitchen now, to the lounge! Very proud!”*, on Day 22. For Florence, her independence came on Day 17 when she reflected, *“If only I didn't need help with the stockings I would have been back to 'normal’ and completely independent”.*

The transition between independence and dependence gradually developed as the desire to return to the “normal self” increased. Before surgery, independence was visible, mainly effected by pain and immobility. During the hospital stay, reliance on others was discernible. Only during the six-week recovery, as progress was made, did the independence return.

## Discussion

This study revealed and interpreted the daily lived experiences of older persons with osteoarthritis, experiencing before, during and after a primary elective total hip or knee replacement in a fast-track programme. All participants had different social and physical circumstances, influencing their perception of how the surgery impacted their daily lives. However, due to the use of diaries, participants had the opportunity to reflect in detail on unique day-to-day feelings and experiences without the possibility of losing significance over time or the influence of the primary author, providing a holistic view of the ‘phenomena’. This study explored the experiences of older persons with osteoarthritis, experiencing an elective total hip or knee replacement in a fast-track programme. Due to the mentally draining effects of pain and the reduced physical mobility caused by OA, social interactions are disrupted when some routine tasks like walking are hindered, therefore influencing the entire person [35]. This was evident from participants’ responses, validating a superordinate theme: the holistic impact of pain on daily quality of life. According to phenotypic features, literature shows the need to integrate multidisciplinary psychological healthcare before surgery for people with pain from OA because of their higher score in pain-associated psychological distress areas [36]. Even for older, vulnerable, and high-risk patients undergoing total hip and knee replacement surgery, improved multimodal and multidisciplinary intraoperative techniques, improved comprehension of postoperative mechanisms of the body, and the application of preventative measures, all result in positive outcomes [5, 8]. In this study, physical and professional interaction formed part of finding ways to cope. Preoperative education programmes provide the advantages of being able to spot exaggerated expectations, discern changes in the social support system, and deal with unforeseen obstacles to home discharge [37]. The findings on psychological and psychosocial support received to cope, is in line with that found in previous research. The need to educate patients and their social networks before surgery and to give anxious patients a chance to talk about their anxieties is another point made by these authors [38]. This would imply that preoperative education may greatly reduce anxiety, despair, and unreasonable expectations, as well as aid people with little social support [37].

In an older population receiving TKR surgery, a recent study indicated that resilience, as a personal trait, was more likely to improve quality of life after surgery [39]. This highlights the necessity of integrating biopsychosocial factors impacting on an individual’s perception in orthopaedic research, emphasizing adjustment and change as a continuous process [40]. Recently, a model for patient health engagement has been suggested in FTPs for thoracic surgery, encouraging increased patient involvement to make the patient's emotional journey more tolerable and to provide them with better coping tools [41]. Focusing on the daily life experiences, this study adds ‘finding ways to cope’ as a superordinate theme. Thoughts of melancholy, worry, and depression have been found to positively correlate with preoperative anxiety, starting 30 min after arrival in the recovery area and lasting up to three days following surgery, according to psychology research [42]. Despite not being utilized as frequently, "faith" was found to be the most effective coping mechanism following colorectal cancer surgery [43].

Essential to FTPs is early mobilization. Numerous studies have discovered advantages such as increased range of motion, muscle strength, and health-related quality of life [44, 45]. Exercise increases older people's functional ability and increases their sense of cognition, self-efficacy and contentment, all of which lead to more independence and happier, more fulfilling lives [46]. According to a study of people who had TKR, the procedure greatly increased their physical, emotional and social activities [47], leading to a more independent lifestyle. This study demonstrated that social, spiritual, emotional, and physical experiences contribute to unique perspectives of the event of a total joint replacement. Pain as a catalyst not only impaired mobilization but triggered emotional roller-coaster feelings during preparation for surgery. Professional and physical support, social relationships, and spirituality were some of the ways participants found ways to cope. During the hospital stay, social adjustments to the new environment were highlighted, generating emotional fluctuations. Participants were constantly finding ways to cope, depending on professional support, using self-reflection and self-motivation, and finding contentment in spirituality. As recovery progressed during the six weeks, the physical impact was gradually accommodating holistic feelings of gratitude and relief. During the recovery period of six weeks, professional support was significant, improving confidence and bringing psychological courage with improved functioning. The findings reinforce the ability of the individual to find a way to cope to defeat adversity, embedded in social and spiritual support.

There is a clear transition between independence before surgery, to reliance on people during hospital stay, and the gradual progress to independent functioning. These findings can guide healthcare professionals to an individualized, person-centred approach. To the best of our knowledge, these findings contribute the first detailed Interpretative Phenomenological Analysis (IPA) account of the transition from independence before surgery, to the dependence during hospital stay, and finally independence gradually obtained during the recovery period. This includes independent walking and return to normal functioning.

### Strengths and limitations

This study aimed to reveal and interpret the experiences of older people with osteoarthritis, experiencing an elective total hip or knee replacement in a fast-track programme. Interpretative phenomenological analysis is considered a strength, the “lived experience” revealing the daily emotional involvement within a psychological phenomenon [48], with roots in phenomenology, hermeneutics and ideography. It is concentrated on describing and interpreting each participant's main experience while being impacted by the researcher's interpretation of each theme [49]. A deliberate decision was made to employ a solicited diary to illuminate individual interpretation in order to learn about and comprehend the lived experiences of participants. To enhance the double hermeneutic process, a reflective journal and reflexive telephonic interviews complemented the diaries. Additionally, to limit the first author’s bias based on a preunderstanding of the orthopaedic practice, continuous and regular peer debriefing sessions during the data-analysis process reflecting on the data and the reflective journal were held between the three authors.

The authors know of no other studies in South Africa with this specific focus on the lived experience as diarized by participants for such an extended period. Based on the ample and elaborate descriptions, participants had time for reflection and sense-making, without interference from the primary author. It should be considered that the study took place in a private hospital setting, which does not represent most South Africans, and people from other socio-economic circumstances may have a different experience. It could be a limitation that participants were represented from only one orthopaedic surgeon’s practice and only one hospital setting. Furthermore, as a nursing professional with a preunderstanding of orthopaedic practice, the possibility of this preunderstanding influencing the data analysis did exist. To minimize the researcher’s bias, telephonic interviews were conducted to augment the data as analysed from the diaries. Concurrently, continuous peer debriefing meetings between the researchers were held to add to the credibility and dependability of the process and ultimately the confirmability of the findings. As five out of the seven participants in this study were women, it cannot be said that gender equality was achieved. The inclusion of only seven participants could be a limitation although small sample sizes is considered reasonable in IPA [15]. Research started pre-COVID and therefore the methodology was adapted during the sampling time of participants to accommodate limited orthopaedic surgery limited. The pathophysiology and contributing variables of osteoarthritis may cause disparities between hip and knee replacements in terms of pain and recovery postoperatively, despite the life experiences of participants undergoing a primary hip or knee replacement being recognized. As observed during the study, the experience of a phenomenon like a replacement surgery had an impact on people's psychological, physical, emotional, and social life. In addition to these, the Covid-19 epidemic during the research period had a greater impact on participant interactions and overall impressions. For some participants the lingering fear of getting COVID-19 while in hospital was mentioned. One was directly influenced by testing positive and surgery had to be postponed while another went through a traumatic event as her husband was hospitalized during the recovery period and she herself became very ill. The absence of supportive relatives during hospitalization as protocols were adapted, added to emotional experiences. Before the Covid-19 pandemic, older person’s experiences probably would have differed.

## Conclusion

Given the effect of OA on the older person, resulting in replacement surgery, this study offered in-depth reflection on participants’ daily experiences, as diarized through these journeys. The three themes highlighted certain important aspects, but fluency is visible in all three stages of the replacement surgery event. Although the benefits of FTP have been nationally and internationally endorsed by literature, the holistic impact of pain, finding ways to cope, and the transition between independence and dependence has not yet been researched in South Africa.

Customizing the general needs of individual patients, building trusting relationships as professionals, and promoting realistic recovery expectations are some of the holistic interventions to be used specifically before surgery, during hospital stay and during recovery. Further research illuminating the intertwined importance of physical, emotional, psychosocial, and spiritual influences during total joint replacement may help address barriers to efficient and improved quality of life after fast-track programmes.

### Supplementary Information

**Additional file 1.**

## Data Availability

On request, the information supporting the study's conclusions is accessible from FW. Data are not publicly accessible due to containing personal and identifying information of the study participants.

## Abbreviations

IPA: Interpretative Phenomenological Analysis
OA: Osteoarthritis
Covid-19: Coronavirus Disease of 2019
FTP: Fast-track programme
THR: Total hip replacement
TKR: Total knee replacement
IP: Independent Person
