# Supplementary material for: A diarized journey: an interpretative phenomenological analysis of the older person’s lived experience of a hip or knee replacement within a fast-track programme
Source: BMC Geriatr. 2023 Sep 25;23:592. doi: 10.1186/s12877-023-04276-4 (PMC10518952; doi:10.1186/s12877-023-04276-4)
Supplement: Supplementary file 1 — Additional file 1. [file 12877_2023_4276_MOESM1_ESM.docx]

**Diary guidelines and questions**

**A Guide for your Diary**

The focus of the study is on how YOU experience you’re hip or knee replacement within the fast-track programme. We are interested in understanding your daily interactions with people, your frustrations or highlights, concerns and discoveries during your hip or knee replacement, in other words, how are you experiencing this time.

Please try to describe your experience as specifically as possible by reflecting on the following aspects:

- Physical experiences (e.g. movement, comfort)
- Emotional experiences (e.g. despair, relief)
- Social experiences (e.g. support,)
- Spiritual experiences (e.g. uplifted, thankful)

Concentrate on specific examples, incidents and or events that come up or stood out during this time. Try to explain why.

Be honest, and reflect on yourself and your feelings. It is ok to experience mixed emotions during this time. Try to reflect on these emotions and experiences and just write down how you feel or felt.

In light of the COVID-19 pandemic, please ensure the diary is only handled by you and placed in a plastic cover and left for 2 days before being given back to the independent person to prevent the spread of the virus.

If you don’t feel up to writing today, there is always tomorrow!

**Diary questions for daily reflections and completion of the diary.**

The following five questions serve as guidance for you to reflect on when you complete your diary. Please try to add entries every day as far as possible. You don’t have to keep to these five questions, you may include as many and all other thoughts and ideas regarding your surgery in this diary. All information will be kept confidential and you will receive the diary back after the research had been completed.

By keeping your hip or knee replacement in mind please complete the following:

1. Describe how you feel today.
   1. How are you feeling physically, emotionally, socially and/or spiritually?
2. Describe any or all events, interactions or incidents that happened today that are related to your replacement surgery (this can include any interactions or discussions with a friend/partner, self-care activities, medical consultations, daily activities, financial concerns etc.)
3. Please write down some of the thoughts and/or concerns that come to mind about your experience of your hip or knee replacement within the fast-track programme. Think about how it influences your future.
   1. What is going through your mind?
   2. How do you feel about things you read or searched on the internet, heard or talked about with friends?
4. How has this replacement surgery impacted your day-to-day life?
5. Please include any comments that you think may contribute to us understanding your experience of your hip or knee replacement within the fast-track programme from your perspective.

You don’t have to complete these questions in the order they are numbered. They are merely a guide, please feel free to reflect and write in a manner that you are comfortable with.
